# Evaluation and Risk Factor Analysis of Post-tonsillectomy Hemorrhage in an Adult Population: An Experience From a National Ear, Nose, and Throat (ENT) Center in Georgia

**DOI:** 10.7759/cureus.68371

**Published:** 2024-09-01

**Authors:** Nino Besiashvili, Irina G Datikashvili-David, Tatia Gakharia

**Affiliations:** 1 School of Natural Sciences and Medicine, Ilia State University, Tbilisi, GEO; 2 Otolaryngology, National Center of Otorhinolaryngology, Japaridze-Kevanishvili Clinic, Tbilisi, GEO; 3 Children's Neurosciences, Tbilisi State Medical University, Tbilisi, GEO

**Keywords:** risk factors, postoperative, complication, adult tonsillectomy, post-tonsillectomy hemorrhage

## Abstract

Background: Tonsillectomy, a common surgical procedure for removing the palatine tonsils, is frequently performed in the otorhinolaryngology department. Tonsillectomy, with or without adenoidectomy, is considered a straightforward operation. However, serious complications, such as post-tonsillectomy hemorrhage, can complicate the recovery period. The research aims to analyze and estimate the factors associated with postoperative bleeding in the adult Georgian population.

Method: We conducted a cross-sectional study. The data was collected retrospectively from the medical records of adult patients aged 18 years and older, who underwent tonsillectomy in 2022 and 2023 at the National Center of Otorhinolaryngology, Japaridze-Kevanishvili Clinic, in Georgia. We performed univariate analysis using binary logistic regression and multivariate logistic regression analysis and calculated odds ratio (OR) to identify factors associated with postoperative bleeding among patients with tonsillectomy. A p-value of <0.05 was considered statistically significant.

Results: A total of 778 adult patients with tonsillectomy were included in the study. Post-tonsillectomy hemorrhage occurred in 14.7% (n=114) of cases, with primary bleeding observed in 8.1% (n=63) of patients and secondary bleeding in 6.6% (n=51) of cases. The highest incidence of bleeding was observed on days 1 (8.1%, n=63) and 7 (1.3%, n=10). The statistical analysis revealed a statistically significant association between post-tonsillectomy hemorrhage and several factors: smoking status (OR=10.1, 95% CI: 6.1-16.7, p<0.001) and having a body mass index (BMI) greater than 25 (OR=3.6, 95% CI: 2.1-6.1, p<0.001).

Conclusion: The study confirmed several significant risk factors, including smoking and higher BMI, that are associated with an increased risk of bleeding among patients, undergoing tonsillectomy. Further research is needed to validate these findings in the Georgian population.

## Introduction

Tonsillectomy is one of the most common surgical procedures performed worldwide. Despite being considered straightforward, it carries risks such as post-tonsillectomy hemorrhage (PTH), which can be life-threatening. The number of tonsillectomies in the United States is estimated at 500,000 procedures annually [1]. Meanwhile, official statistical data for Georgia is unavailable.

The recovery period of tonsillectomy can have serious complications such as pulmonary edema, dehydration, and bleeding; in some cases, blood transfusions are required [2]. PTH may occur at any time (within postoperative days 0-21), and it is classified as primary and secondary [3]. Primary hemorrhage is defined as postoperative bleeding which occurs within the first 24 hours of surgery, while bleeding after 24 hours is classified as secondary. Although secondary bleeding is less frequent [4], PTH is more common in adults than in patients under 18 years old. However, PTH risk factors and preventive measures are unclear.

This surgery is performed on patients of all ages, ranging from children to adults. Tonsillectomy is performed to remove both palatine tonsils. Various techniques include cold steel dissection, electrocautery, laser ablation, and harmonic scalpel. These methods differ in their potential benefits, allowing surgeons to plan operations based on patient characteristics and preferences [5,6]. In Georgia, the most frequent methods of tonsillectomy are laser and bipolar techniques.

The prevalence of PTH among surgeons and patients is substantial. Many researchers posit that the rate of PTH depends on the geographical location [7]. However, there is a lack of data evaluating PTH in the Georgian population.

This study aims to identify the factors associated with PTH in Georgia.

## Materials and methods

This cross-sectional study was conducted at the National Center of Otorhinolaryngology, Japaridze-Kevanishvili Clinic, a leading facility specializing in ear, nose, and throat (ENT) surgeries in Georgia. The clinic serves a diverse patient population and has a capacity for handling a large number of surgical procedures.

Study participants were patients aged 18 and older who underwent tonsillectomy between December 2022 and July 2023. The study adhered to international ethical standards and was approved by the Ilia State University Medical Faculty and Institutional Review Board (approval number: R/241-23). Patient confidentiality was strictly maintained, and all data were anonymized.

Variables and data collection

Patient data were collected retrospectively from medical records. The variables included in the analyses are as follows: age, gender, history of recurrent tonsillitis, presence of peritonsillar abscess, smoking history (current vs. never smokers), and body mass index (BMI). Surgical data encompassed the date of secondary hemorrhage, the incidence of recurrent bleeding, the surgical technique used (bipolar or laser dissection), the surgeon's experience (junior vs. senior doctor), and the season during which the surgery was performed (cold season: December-February; warm season: March-July). Postoperative use of non-steroidal anti-inflammatory drugs (NSAIDs, ibuprofen or paracetamol) was also collected. Patients were stratified into two age groups: 18-30 years and older than 30 years.

Surgical procedures

All surgeries were performed under general anesthesia. Tonsillectomies were performed using either bipolar dissection or laser dissection techniques. The choice of technique depended on the surgeon's preference and the specific clinical scenario. Detailed operative notes were reviewed to extract this information.

Postoperative care

Postoperative care included standardized protocols for pain management and monitoring for complications. The use of NSAIDs during the postoperative period was documented, with specific attention to ibuprofen and paracetamol usage.

Data analysis

For descriptive statistics, categorical variables were presented as numbers and percentages (n, %). Continuous variables were expressed as mean±standard deviation (SD). To identify factors associated with postoperative bleeding among patients undergoing tonsillectomy, we performed univariate analysis and multivariate logistic regression analysis and calculated odds ratio (OR) with 95% of confidence interval (95% CI). The factors, which showed a statistically significant correlation with PTH in univariate analysis, were included in a multivariate model. A p-value of <0.05 was considered statistically significant. Statistical analyses were performed using IBM SPSS Statistics for Windows, V. 25.0 (IBM Corp., Armonk, NY).

## Results

A total of 778 adult patients who underwent bilateral tonsillectomy were included in the study. Of these, 50.5% (n=393) were males, and the mean age was 27 (±8.0) years. The prevalence of PTH was 14.7% (n=114), and primary bleeding occurred in 8.1% (n=63) of patients. Table 1 provides the general descriptive characteristics of the study participants and possible risk factors for PTH.

**Table 1:** Basic characteristics of the study participants and risk factors for PTH. PTH: post-tonsillectomy hemorrhage; BMI: body mass index; NSAIDs: non-steroidal anti-inflammatory drugs

Risk factors for PTH	N (%)
Gender	
Male	393 (50.5)
Female	385 (49.5)
Age, mean (SD)	28.7 (±8.0)
Techniques used for tonsillectomy	
Bipolar	528 (67.9)
Laser	250 (32.1)
History of peritonsillar abscess (at least once in their life)	
Yes	91 (11.7)
No	687 (88.3)
BMI	
Underweight	117 (15)
Normal weight	528 (67.9)
Overweight	132 (17)
Obesity	1 (0.1)
Hemorrhage	
Non-PTH	664 (85.3)
PTH	114 (14.7)
PTH	
Primary	63 (8.1)
Secondary	51 (6.56)
Season of operation	
Cold	264 (33.9)
Warm	514 (66.1)
Experience of the surgeon	
Junior doctor	174 (22.4)
Senior doctor	604 (77.6)
Intake of NSAIDs during the postoperative period	
Paracetamol	221 (28.4)
Ibuprofen	557 (71.6)
History of smoking	
Smokers	155 (19.9)
Non-smokers	623 (80.6)
History of recurrent tonsillitis	
Yes	673 (86.5)
No	105 (13.5)

**Figure 1:**
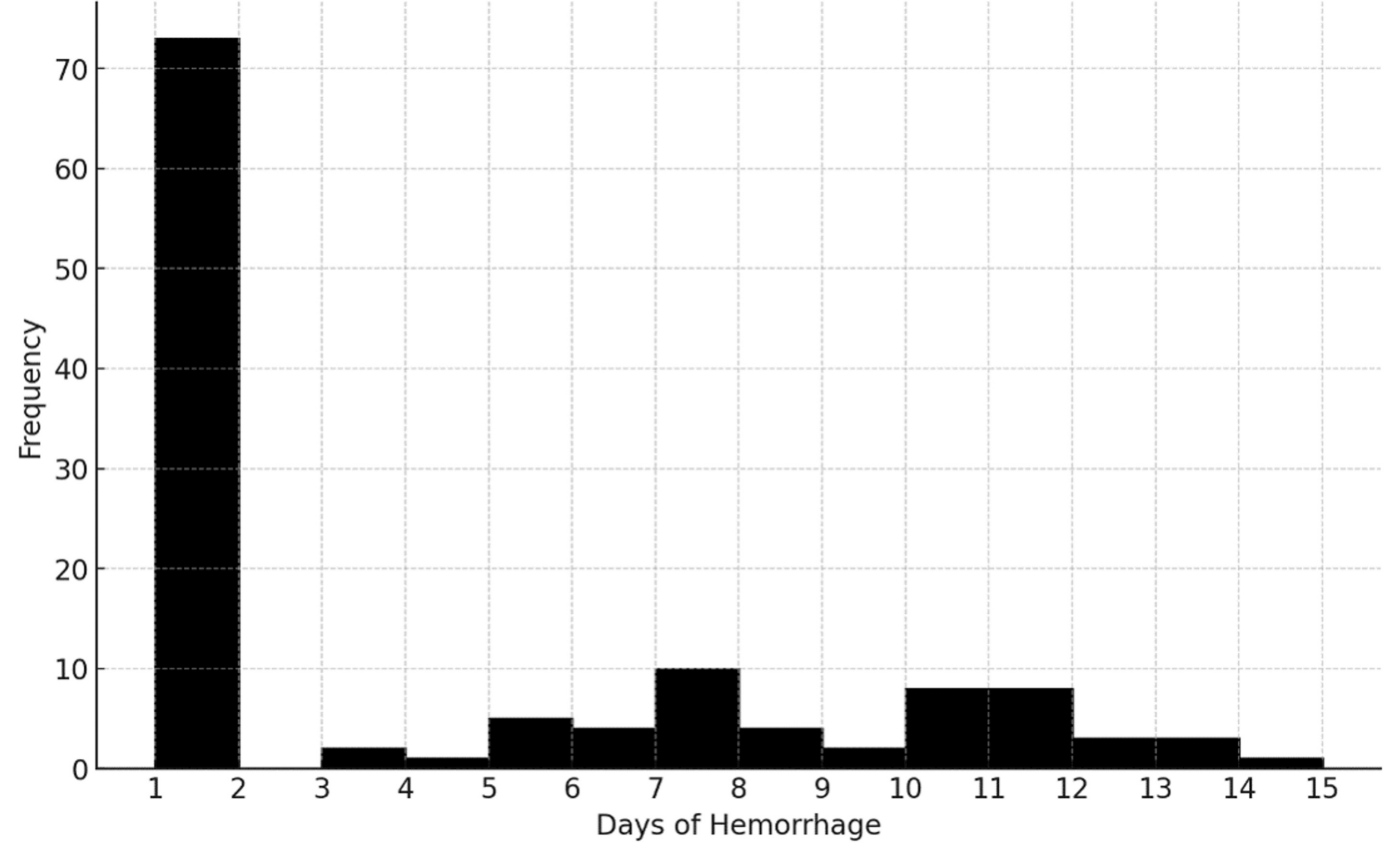
Frequency of PTH days. PTH: post-tonsillectomy hemorrhage

Bleeding was observed mainly on the first (8.1%, n=63/778) and seventh (1.3%, n=10/778) days after surgery (Figure 1).

The chi-squared test showed several factors significantly associated with PTH (Table 2). These factors included smoking status (p<0.001), BMI greater than 25 (p<0.001), season (p<0.001), method of operation (p<0.001), history of recurrent tonsillitis (p=0.002), and history of peritonsillar abscess (p=0.016). However, gender, age, the surgeon's experience, and the use of NSAIDs during the postoperative period were less likely to be risk factors for PTH.

**Table 2:** The impact of various risk factors on PTH. *Significant at p≤0.05 level. PTH: post-tonsillectomy hemorrhage; BMI: body mass index; NSAIDs: non-steroidal anti-inflammatory drugs

Variables	Non-PTH	PTH	P-value
Age groups (years)			
30 and younger	430 (85.5%)	73 (14.5%)	0.881
31 and older	234 (85.1%)	41 (14.9%)	
Gender			
Male	331 (84.2%)	62 (15.8%)	0.371
Female	333 (86.5%)	52 (13.5%)	
Experience of the surgeon			
Junior	149 (85.6%)	25 (14.4%)	0.904
Senior	515 (85.3%)	89 (14.7%)	
Season			
Warm	479 (93.2%)	35 (6.8%)	<0.001*
Cold	185 (70.1%)	79 (29.9%)	
NSAIDs in the postoperative period			
Ibuprofen	474 (85.1%)	83 (14.9%)	0.756
Paracetamol	190 (86.0%)	31 (14.0%)	
Method of the operation			
Laser	194 (77.6%)	56 (22.4%)	<0.001*
Bipolar	470 (89.0%)	58 (11.0%)	
BMI			
Below 25	583 (90.4%)	62 (9.6%)	<0.001*
25 and above	81 (60.9%)	52 (39.1%)	
Smoking			
Yes	84 (12.7%)	71 (45.8%)	<0.001*
No	580 (93.1%)	43 (6.9%)	
History of recurrent tonsillitis			
Yes	564 (83.8%)	109 (16.2%)	0.002
No	100 (95.2%)	5 (4.8%)	
History of peritonsillar abscess (at least once in anamnesis)			
Yes	70 (76.9%)	21 (23.2%)	0.016
No	594 (86.5%)	93 (13.5%)	

Most of the surgeries (66.1%, n=514) were conducted during the warm season. Based on the chi-squared test, patients operated on during this period had a higher risk of developing PTH compared to those operated on during the cold season. However, this association is not statistically significant based on the multivariate logistic regression (OR=0.2, 95% CI: 0.1-0.3, p<0.001) (Table 3).

**Table 3:** Multivariate logistic regression analysis of the impact of various risk factors on PTH. P<0.05 is considered statistically significant. Ref: reference category; PTH: post-tonsillectomy hemorrhage; BMI: body mass index

Factors	Adjusted odds ratio	95% CI	P-value
Method of the operation			
Bipolar	Ref		
Laser	0.65	0.392-1.077	0.095
Season of the operation			
Cold	Ref		
Warm	0.188	0.111-0.316	<0.001
BMI			
Below 25	Ref		
25 and above	3.584	2.107-6.096	<0.001
History of peritonsillar abscess (at least once in their life)			
Yes	Ref		
No	0.41	0.209-0.804	0.01
History of recurrent tonsillitis			
Yes	Ref		
No	0.188	0.65-0.546	0.002
Smoking status			
Yes	Ref		
No	10.058	6.073-16.657	<0.001

Multivariate analysis confirmed that smoking (OR=10.1, 95% CI: 6.1-16.7, p<0.001) and having a BMI greater than 25 (OR=3.6, 95% CI: 2.1-6.1, p<0.001) were independent predictors of PTH. However, patients with a history of peritonsillar abscess (OR=0.4, 95% CI: 0.2-0.8, p=0.010) and recurrent tonsillitis (OR=0.2, 95% CI: 0.7-0.5, p=0.002) were less likely to experience bleeding post-tonsillectomy (Table 3). Additionally, the odds of bleeding were lower for the laser method (OR=0.7, 95% CI: 0.4-1.0, p=0.095) compared to bipolar.

Other factors such as age, gender, surgical technique, and surgeon's experience were not significantly associated with PTH after adjusting for confounders.

## Discussion

Tonsils are highly vascularized organs; consequently, bleeding from this area in the postoperative period is a significant concern for clinical practitioners and patients. However, PTH's risk factors and prevention mechanisms remain unclear [8]. Our cross-sectional study indicates that smoking and higher BMI are significant predictors of PTH.

Our study found that the majority of patients experienced PTH within the first week following the surgery. Primary bleeding was more common than secondary (day 7). An observation is that the rate of PTH varies across different studies. A study conducted on 3,306 pediatric and adult patients showed a hemorrhage rate of 1.78%, with all cases classified as secondary PTH [9]. In contrast, the prevalence of PTH in the Georgian adult population was higher (14.7%). 

We found that smoking status was a significant predictor of PTH. The higher prevalence of smoking in Georgia (26.4% of the population) may contribute to the increased rate of PTH observed in our study [10]. Nearly half of the patients who experienced PTH had an active smoking status. In the past, another retrospective study identified a significant connection between smoking and recurrent tonsillitis. However, it was not considered as one of the main risk factors of PTH [11]. Moreover, evidence indicates that smoking has a detrimental impact on wound healing and increases the risk of postoperative bleeding in general [12-15]. 

Numerous studies have identified obesity (BMI>25) as an independent risk factor for the incidence of PTH [8]. Our data support these findings (OR=3.93).

NSAIDs impair platelet aggregation and can contribute to bleeding. Numerous studies have examined the effect of NSAIDs on PTH. A retrospective cohort study of 1,057 adult patients found no association between NSAID use and the rate of PTH [16]. However, in the pediatric population, another study identified NSAIDs as a risk factor for PTH [17]. In our study, we did not find significant differences between the intake of ibuprofen and paracetamol concerning bleeding risk.

Several studies have identified recurrent tonsillitis as a major predictor of PTH during the postoperative period, with most cases of bleeding attributed to improper healing [18]. Our data showed that a quarter of patients with a history of PTH had previously experienced a peritonsillar abscess. A similar association was observed between recurrent tonsillitis and PTH in our analysis. However, multivariate logistic regression revealed that these two factors are not strong predictors of PTH.

Some studies identified gender as a significant risk factor for PTH, probably due to the effects of female hormones like estrogen on wound healing [19]. However, our study found no difference between the groups concerning the patient's sex, although age is commonly identified as a risk factor for hemorrhage, increasing the risk in older patients [4]. This study found no significant difference between the two groups. Contrary to expectations, no association was found between PTH and surgical approach or surgeon's experience.

Despite the limitations, this study was the first cross-sectional study about PTH in this region. It can be the baseline for future researchers to validate these potential risk factors in our region.

Limitations

There are limitations to this study. First, the data collection period was brief and not diverse. We have collected the data from only one center which has the highest number of patients in Georgia; thus, the sample size could be considered small. It could be better to cover and represent these findings with large cohorts. Second, we believe there may have been residual confounding. Despite our efforts to control various known confounding factors, it is possible that other unmeasured or unknown variables could have influenced the results. Third, there could be missing data for those patients who had bleeding that was stopped spontaneously and were not hospitalized.

## Conclusions

The study confirmed significant associations between several predictors and PTH, including BMI and smoking. These findings highlight the importance of considering patient-specific and environmental factors when assessing hemorrhage risk. The results align with the existing evidence and highlight the need for region-specific preventive measures.
